# Therapeutic effect of biosynthetic gold nanoparticles on multidrug-resistant *Escherichia coli* and *Salmonella* species isolated from ruminants

**DOI:** 10.14202/vetworld.2021.3200-3210

**Published:** 2021-12-29

**Authors:** Abeer M. Abdalhamed, Alaa A. Ghazy, Eman S. Ibrahim, Amany A. Arafa, Gamil S. G. Zeedan

**Affiliations:** 1 Department of Parasitology and Animal Diseases, National Research Centre, Dokki, Egypt; 2 Department of Microbiology and Immunology, National Research Centre, Dokki, Egypt

**Keywords:** *Escherichia coli*, gold nanoparticle, multidrug-resistant, ruminant, *Salmonella* species

## Abstract

**Background and Aim:**

Multidrug-resistant (MDR) pathogenic microorganisms have become a global problem in ruminants as a result of the intensive use of antibiotics, causing the development of resistance among gut microbiota. The antibiotic-resistant microorganisms can be transferred from diseased animals to humans. This study aimed to determine the prevalence of MDR *Escherichia coli* and *Salmonella* spp. isolated from cattle, buffaloes, sheep, and goats suffering from respiratory signs, diarrhea, and mastitis and to screen the antibiotic sensitivity of selected isolated bacteria. It also detected antibiotic-resistance genes by polymerase chain reaction (PCR), produced green gold nanoparticles (AuNPs) using plant extracts (*Artemisia herba-alba* and *Morus alba*), and evaluated the antimicrobial activities of these biosynthesized nanoparticles on selected pathogens (*E. coli* and *Salmonella* spp.).

**Materials and Methods:**

MDR *E. coli* and *Salmonella* spp. were investigated using fecal samples (n=408), nasal swabs (n=358), and milk samples (n=227) of cattle, buffaloes, sheep, and goats with or without clinical signs, including respiratory manifestations, pneumonia, diarrhea, and mastitis, from different governorates in Egypt. *E. coli* and *Salmonella* spp. were isolated and identified on selective media, which were confirmed by biochemical reactions and PCR. Antimicrobial susceptibility testing against 10 commonly used antibiotics was performed using the Kirby-Bauer disk diffusion method. Antibiotic resistance genes *bla_TEM,_ bla_SHV,_ bla_OXA_*, and bla*_CTX−M_* were detected by PCR. The antibacterial effect of the biosynthesized AuNPs was evaluated by MIC and well diffusion assay. The biosynthesized AuNPs were also characterized by ultraviolet-visible spectrophotometry and transmission electron microscopy (TEM).

**Results:**

Among all fecal samples, the prevalence of *E. coli* was 18.4% (183/993) and that of *Salmonella* spp. was 16.7% (66/408), as determined by cultural and molecular tests. All isolates of *E. coli* and *Salmonella* spp. were 100% resistant to ampicillin (AM) and amoxicillin and highly resistant to cefoxitin and AM-sulbactam. The total rate of resistance genes in *E. coli* was 61.2% (112/183), while that in *Salmonella* was 63.6% (42/66) for pathogens isolated from ruminants with respiratory manifestations, pneumonia, diarrhea, and mastitis. Among the resistance genes, *bla_TEM_* had the highest prevalence rate in *E. coli* (25.9%, 21/81) while *bla_SHV_* had the lowest (9.8%, 8/81) in fecal swabs. AuNPs were successfully synthesized using aqueous leaf extract of *A. herba-alba* and *M. alba* as bioreducing agents. TEM analysis showed particle size of 10-42 nm for *A. herba-alba* and *M. alba* AuNPs. The biosynthesized AuNPs showed antibacterial activity against MDR *E. coli* and *Salmonella* spp.

**Conclusion:**

Rapid and accurate diagnostic methods are the cornerstone for effective treatment to reduce the risk of antimicrobial-resistant pathogenic microorganisms. This is particularly important for overcoming the increasing rate of MDR in ruminants with respiratory manifestations, pneumonia, diarrhea, and mastitis. This can be complemented by the development of AuNPs synthesized in an environmentally friendly manner AuNPs using natural plant extracts for the treatment of antibiotic-resistant microorganisms.

## Introduction

The prevalence of multidrug resistance (MDR) is increasing among various pathogenic bacteria worldwide, leading to antimicrobials no longer being effective for controlling infectious diseases [1]. MDR bacteria are a global problem in ruminants due to the intensive use of antibiotics as growth promoters and for the prevention and treatment of disease [2,3]. The presence of resistant pathogenic strains in dairy cattle, calves, beef cattle, sheep, goats, and feedlot animals constitute the primary reservoir of zoonotic pathogens. Emergent strains of MDR bacteria can be transferred from ruminants to humans through food consumption (meat and milk), or through direct or indirect contact with animals or their waste in the environment [4]. *Escherichia coli* strains isolated from animals are resistant to commonly used antibiotics, which acquire resistance through horizontal gene transfer or gene mutations. They are a major cause of foodborne infections and commonly inhabit the gastrointestinal tract of animals and humans [5]. *Salmonella* is commonly responsible for enteric bacterial infection of large and small animals, but such infection is more severe in infants from the first 2 weeks to 3 months of life. *E. coli* and *Salmonella* species are among the most common and economically damaging pathogens causing diarrhea in neonates of animals used by humans for food [6].

Globally, the β-lactam antibiotics (cephalosporin, carbapenem, and penicillin) represent about 60% of all antimicrobial agents used in animals. Their habitual abuse has led to the development of resistance to beta-lactam groups [7]. The recent emergence of MDR *E. coli* poses a major threat to public health, which is mainly attributed to extended-spectrum beta-lactamases (ESBLs). These enzymes can destroy various β-lactam antimicrobial agents and are encoded by specific ESBL genes, such as *bla_TEM_* (encoded for penicillin resistance), *bla_KPC_* (encoded for carbapenems resistance), and *bla*_CTX_ (encoded for cephalosporin resistance). The emergence of MDR virulent *E. coli* has become a global health concern [8,9]. Most of the β-lactam resistance in *Salmonella* is provided by horizontally acquired β-lactamases. However, many other bacteria have intrinsic ampC, *bla_TEM_*, *bla*_CTX-M_, *bla_IMP_*, *bla_VIM_*, *bla_KPC_*, *bla_SHV_*, and *bla_OXA_* [10]. MDR strains such as *Salmonella* spp. and *E. coli* are not susceptible to many antibiotics, so alternative treatments using green nanoparticles (NPs) are required to eliminate them [11].

The green synthesis of NPs has attracted substantial interest among scientists. Plant extracts are a promising tool for the green synthesis of NPs, given their convenience, ease of use, environmentally friendly nature, and the minimization of the harmful biohazardous effects of chemical and physical methods, along with the avoidance of toxic chemicals and dangerous by-products. Green-synthesized NPs have biocidal effects against Gram-positive and Gram-negative bacteria that cause different diseases in animals [12,13]. Moreover, antibiotics selectively target bacteria, while gold and silver NPs exhibit fairly broad-spectrum activity. AuNPs act by destroying the cell membrane or by inhibiting the binding of tRNA to the small subunit of the ribosome, leading to the suppression of protein synthesis [14]. It has also been reported that NPs display effective inhibitory activity against resistant strains such as ampicillin (Am)-resistant *E. coli* [15]. Given their stability and antibacterial activity, synthesized gold NPs (AuNPs) are promising for biomedical applications [16]. Effective treatment of MDR bacteria requires the rapid and accurate diagnosis of their presence using phenotypes, along with molecular methods to detect the presence of resistance genes [17]. A number of novel approaches for the use of green NPs to combat bacterial resistance are currently available [18].

The present study aimed to evaluate the therapeutic effect of biosynthetic AuNPs developed using a mixture of *Artemisia herba-alba* and *Morus alba* leaf extract against MDR *E. coli* and *Salmonella* spp. isolated from ruminants (cattle, buffaloes, sheep, and goats) with clinical signs including respiratory manifestations, pneumonia, diarrhea, and mastitis.

## Materials and Methods

### Ethical approval

All methods were performed in accordance with relevant guidelines and regulations. Well-trained experts conducted the handling of animals and experimental procedures. The handling of animals and all protocols were approved by the Animal Ethics Review Committee of National Research Centre, Egypt, under approval number #19-149#.

### Study period, area, and sample collection

The study was conducted from December 2019 to March 2021. A total of 358 nasal and 408 fecal swab samples and 227 milk samples were collected from cattle, buffaloes, sheep, and goats in different governorates in Egypt (Monofia, Giza, Beni-Suef, Alexandria, Siwa, and Marsa Matruh). This included animals with or without clinical signs, including respiratory manifestations, pneumonia, diarrhea, and mastitis. Nasal swabs were collected (n=358) from cattle (n=38), buffaloes (n=40), sheep (n=155), and goats (n=125). Meanwhile, fecal samples (n=408) were collected from cattle (n=52), buffaloes (n=116), sheep (n=135), and goats (n=105). Moreover, milk samples (n=227) were collected from cattle (n=54), buffaloes (n=36), sheep (n=62), and goats (n=75). Nasal and rectal/fecal swabs collected in sterile sample tube contained sterile saline or PBS. The samples were transported in ice box and stored between 4°C-8°C for up to 72 hours after collection and milk sample was divided into 2 aliquots: the first was plated fresh for bacteriological culture, to isolate and identify *E. coli* and *Salmonella*., While, the second milk sample was frozen at −20°C for bacteriological culture, and DNA extraction for molecular diagnosis. All samples were subjected to detect resistance genes.

### Isolation and identification of *E. coli*

All samples (358 nasal swab, 408 fecal swab, and 227 milk samples) collected from cattle, buffaloes, sheep, and goats with clinical signs including respiratory manifestations, pneumonia, and diarrhea were placed on peptone water broth (Oxoid, UK) as pre-enrichment for bacterial growth, in accordance with the methods described previously [19]. A loopful of culture inoculate was streaked on MacConkey (Oxoid) agar. Pink colonies obtained from MacConkey agar that was indole-positive were considered positive for *E. coli* and maintained at −20°C in 20% glycerol brain heart infusion broth for further confirmation and characterization by polymerase chain reaction (PCR) and antibiotic susceptibility testing, in accordance with a previous study [20].

### Isolation and identification of *Salmonella* spp.

Fecal swabs were collected from cattle, buffaloes, sheep, and goats with clinical signs including respiratory manifestations, pneumonia, and diarrhea (n=408) and pre-enriched on BPW (Oxoid), incubated at 37°C for 16 h. One milliliter of inoculum was transferred into selenite cystine broth (Oxoid) after pre-enrichment, as described previously [21]. A loopful of inoculum was also plated onto xylose lysine deoxycholate (XLD) (Oxoid) medium and incubated at 37°C for 24 h. Each colony with a black center from XLD was inoculated in brilliant green agar (Oxoid) and incubated. Triple sugar iron, urease, and citrate tests were performed for the confirmation of *Salmonella* spp. and further confirmation and characterization were carried out by PCR and antibiotic susceptibility testing, as described previously [22].

### DNA extraction by different methods

DNA extraction was carried out by different heating methods (boiling, microwaving, and heat block). Briefly, a pure colony collected from freshly grown culture was initially placed in an Eppendorf tube containing molecular-grade water (100 mL) followed by mixing gently through vortexing. Subsequently, the mixture was heated for 10 min, cooled for 10 min, and centrifuged for 10 min at 1400 rpm. Finally, the supernatant was collected as the source for the genomic DNA for PCR and stored at −20°C until further use.

The PCR reaction was carried out in a final volume of 25 μL with 12.5 μL of master mix (2×) (Promega, Madison, WI, USA), 4 μL of genomic DNA (50 ng/μL), 1 μL of each primer, and 6.5 μL of nuclease-free water. After amplification, PCR products were subjected to gel electrophoresis in 1.5% agarose, followed by staining and visualization with 0.25% ethidium bromide solution and an ultraviolet transilluminator. A DNA ladder (100 bp; Promega) was used to assess the sizes of the PCR amplicons.

### Molecular confirmation of isolated *E. coli* and *Salmonella* spp.

Isolated *E. coli* and *Salmonella* spp. were confirmed by PCR targeting an *E. coli* (*LTI)* gene and a *Salmonella* genus-specific (*fmA*) gene, as shown in Table-1, in accordance with a previous study [23].

**Table 1:** List of primers used for detecting *E. coli* and *Salmonella* spp.

Isolated bacteria	Target genes	Primer sequence (5ʹ→3ʹ)
*Salmonella* spp.	*fmA*	(F) 5 -CCT TTC TCC ATC GTCCTG AA-3, (R) 5 -TGC TGT TAT CTG CCT GAC CA-3
*E. coli*	*LTI*	(F) 5-AGCAGGTTTCCCAC CGGATCACCA-3, (R) 5-GTGCTCAGATTCTGGGTCTC-3

*E. coli=Escherichia coli*

### Antimicrobial sensitivity test (AST)

The isolated *E. coli* and *Salmonella* spp. were subjected to AST using the disk diffusion technique, in accordance with the guidelines of the Clinical and Laboratory Standards Institute [24,25]. The testing involved 10 commonly used antibiotics, including three groups: A quinolone group (levofloxacin: LEV [5 μg/disk] and ciprofloxacin: CIP [5 μg/disk]); a beta-lactam group (amoxicillin: Ax [25 μg/disk], Am [10 μg/disk], cefoxitin: CX [30 μg/disk], imipenem: IMP [10 μg/disk], cefotaxime: CTX [30 μg/disk], and Am/sulbactam: SAM); and an aminoglycoside group (gentamicin: CN [10 μg/disk], [20 μg/disk], and amikacin: AK [30 μg/disk]), following the disk diffusion method described previously [25]. Finally, the zone of growth inhibition was compared using standards provided elsewhere [26] to identify the resistant isolates. Isolates showing resistance to three or more different classes of antibiotics were defined as MDR [27].

### Detection of resistance genes in MDR *E. coli* and *Salmonella* spp. isolated from diseased animals by PCR

The status of bacteria with multidrug resistance against beta-lactams (Am, Ax, Am/sulbactam, and CX) as determined by AST was further confirmed by the detection of resistance genes (*bla_TEM_*, *bla_SHVm_*, *bla_OXA_*, and *bla_CTX−M_*) using PCR, in accordance with a previous study [28], using the resistance gene primers listed in Table-2.

**Table 2:** Oligonucleotide primers of β-lactamase resistance genes of isolated *E. coli* and and *Salmonella* spp.

Genetic resistance factors	Genes	Primer sequence (5ʹ→3ʹ)	Size (bp)
β-lactam genes (*bla*)	*bla_TEM_*	F: ATG AGT ATT CAA CAT TTC CG R: CCA ATG CTT ATT CAG TGA GG	1080
	*bla_SHV_*	F : TTA TCT CCC TGT TAG CCA CC R: GAT TTG CTG ATT TCG CTC GG	768
	bla_OXA_	F: ATG AAA AAC ACA ATA CAT ATC R: AAT TTA GTG TGT TTA GAA TGG	813
	bla*_CTX−M_*	F: -ATG TGC AGY ACC AGT AAR GT R: -TGG GTR AAR TAR GTS ACC AGA	544

*E. coli=Escherichia coli*

### Preparation of *A. herb-alba* and *alba* aqueous leaf extracts

The preparation of leaf extracts was carried out in accordance with a previous study [29]. *A. herba-alba* and *M. alba* leaves were washed using double-distilled water, air-dried, and then each dried plant was ground separately into a fine powder. Briefly, 10 g of powder from each of *A. herba-alba* and *M. alba* was dissolved in 100 mL of double-distilled water in a conical flask. The solution was boiled at 70-80°C for 15-20 min in a water bath. The solution was then filtered at room temperature (25oC) and stored at 4°C for further analysis.

### Biosynthesis of AuNPs

AuNPs were synthesized using *A. herba-alba* and *M. alba* leaf extracts. Specifically, 10 μL of *A. herba-alba* and *M. alba* leaf extract (v/v) was mixed with 90 μL of 1 mM aqueous solution of gold chloride (HAuCl_4_) and left at 25oC to react. After 20 min, a change in the color of the solution from yellow to purple/red was identified, which signified the reduction of Au^3+^ to Au^0^ NPs. The resulting AuNPs were incubated at 25^o^C for 24 h and then purified by centrifugation at 10080 x g for 15 min. The pellet was purified by several washes in distilled water and then transferred to a Petri dish for drying overnight at 30°C.

### Characterization of AuNPs

The wavelength with maximum absorption of the obtained NPs was measured by UV-spectra. The size distribution and shape of the colloidal AuNPs were studied by SEM and transmission electron microscopy (TEM).

### Antimicrobial assay for AuNPs

The antimicrobial activity was analyzed using the agar well diffusion method [30]. The samples were maintained in broth and subcultured in a Petri dish before testing. First, 100 μL of *A. herba-alba* and *M. alba* AuNPs were loaded into each well on Mueller-Hinton (MH) agar plates. These plates were subsequently sealed and incubated face upwards in an incubator at 37±0.2°C for 24 h. The appearance of a clear/white area around the wells was recorded and measured (in millimeters).

### Growth of bacteria at different concentrations of biosynthetic AuNPs

*E. coli* and *Salmonella* suspensions (0.2 mL) were inoculated into corresponding tubes containing 1.5 μL of different concentrations of *A. herba-alba*, *M. alba*, and *A. herba-alba*+*M. alba* AuNPs, and 1.5 μL of MHB. To these test tubes, 1 μL of phenol red indicator solution was added. Tubes containing inoculum alone served as positive controls and tubes with different biosynthetic NPs+nutrient media served as negative controls. Test tubes with only MHB served as a blank control. The tubes were incubated at 37°C for 24 h and were observed for changes in color and pH.

### Statistical analysis

SPSS software version 20.0 (IBM, USA) was used to analyze the data. Frequency and mean were estimated using descriptive analysis.

## Results

### Prevalence of *E. coli* and *Salmonella* spp. in investigated animals

Isolates of *E. coli* and *Salmonella* spp. from animals with respiratory manifestations, pneumonia, diarrhea, and mastitis were identified and characterized based on their morphology, biochemically identified, and confirmed by molecular assays. The bacteriological analysis included microscopic analysis. In this analysis, the bacterial isolates that were Gram-negative bacilli of moderate size and produced pink colonies on MacConkey agar were designated as *E. coli*, while the black-centered colonies on XLD were designated as *Salmonella* spp. All *E. coli* isolates were identified by biochemical tests, showing positivity in catalase, lactose fermentation, indole, and methyl red tests. They were also negative in cytochrome oxidase, Voges–Proskauer, citrate utilization, H_2_S production, and urease tests. The bacteriological analysis and molecular assays showed positivity for *E. coli* in 18.4% (183/993) of all samples. The prevalence of *E. coli* was 5.02% (18/358) from nasal samples of the different animals, while it was 19.8% (81/408) from fecal swabs and 37% (84/227) from milk samples. Meanwhile, the total prevalence of *Salmonella* spp. in fecal swabs collected from different animals was 16.17% (66/408). In fecal samples, the highest prevalence of *Salmonella* spp. was 26.9% (14/52) in cattle, while the lowest was 10.34% (12/116) in buffaloes, as shown in Table-3.

**Table 3:** Prevalence of *E. coli* and *Salmonella* spp. in different animals.

Samples	Species	No. of samples	*E. coli*		*Salmonella* spp.	
	
			+ve	%	+ve	%
Nasal samples	Cattle	NS (n=38)	2	5.2		
	Buffaloes	NS=(40)	2	5		
	Sheep	NS=(155)	6	3.8		
	Goats	NS=(125)	8	6.4		
	Total	358	18	5.02		
Fecal samples	Cattle	FS=(52)	11	21.1	14	26.9
	Buffaloes	FS=(116)	22	18.9	12	10.34
	Sheep	FS=(135)	23	17	22	16.29
	Goats	FS=(105)	25	23.8	18	17.14
	Total	408	81	19.8	66	16.17
Milk samples	Cattle	Milk=(54)	16	29.6		
	Buffaloes	Milk=(36)	16	44.4		
	Sheep	Milk=(62)	21	33.8		
	Goats	Milk=(75)	31	41.3		
	Total	227	84	37		
Total isolates		993	183	18.4	66	16.17

NS=Nasal swab, FS=Fecal swab, MS=Milk sample. *E. coli=Escherichia coli*

### Detection of beta-lactam resistance genes by PCR

**Figure-1:**
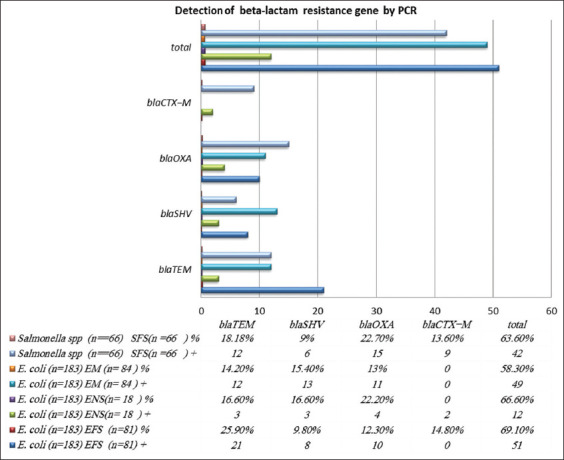
Detection of beta-lactam resistance genes by polymerase chain reaction (PCR). EFS=*Escherichia coli* isolated from fecal samples. ENS=*Escherichia coli* isolated from nasal swabs. EMS=*Escherichia coli* isolated from milk samples. SFS=*Salmonella* spp. isolated from fecal samples.

**Figure-2:**
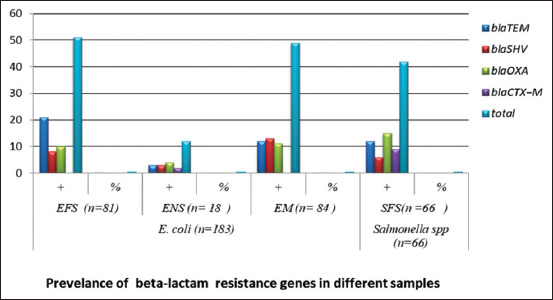
Prevalence of beta-lactam resistance genes in different samples. EFS=*Escherichia coli* isolated from fecal samples. ENS=*Escherichia coli* isolated from nasal swabs. EMS=*Escherichia coli* isolated from milk samples. SFS=*Salmonella* spp. isolated from fecal samples.

The isolated *E. coli* (n=183) and *Salmonella* spp. (n=66) were screened by PCR to detect antibiotic resistance genes. The total resistance to *E. coli* and *Salmonella* spp. detected by PCR was 61.2% (112/183), for beta-lactam genes in *E. coli* and *Salmonella* was 63.6%. (42/66). The highest percent o*f bla_TEM_* resistance genes had the highest prevalence, which was present at a rate o*f* 25.9% (21/81), while the lowest rate was for *bla_SH_*V, present at a rate of 9.8% (8/81), as shown in Figures-1 and 2.

### Biosynthesis of *A. herba-alba* and *M. alba* AuNPs

**Figure-3:**
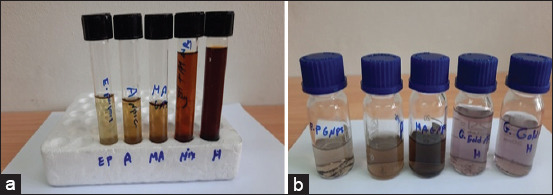
(a and b) Observation of the color changes confirming AuNP formation in the reaction mixture at different time points from left to right: Pale yellow, light pink, light red and dark red, dark brown, and purple. The successful biosynthesis of *Artemisia herb-alba* and *Morus alba* AuNPs was achieved by adding a mixture of *A. herba-alba* and *M. alba* leaf extract to an aqueous solution of gold chloride (HAuCl_4_), as indicated by a change in solution color from yellow to purple/dark red and dark brown, signifying the reduction of Au^3+^ to Au^0^ NPs.

The successful biosynthesis of *A. herba-alba* and *M. alba* AuNPs was achieved by adding a mixture of *A. herba-alba* and *M. alba* leaf extract to an aqueous solution of gold chloride (HAuCl_4_). This was indicated by a change in the color of the solution from yellow to purple/dark red and dark brown, signifying the reduction of Au^3+^ to Au^0^ NPs (Figure-3).

### Ultraviolet–visible (UV–Vis) spectral analysis

The reduction of aqueous HAuCl_4_ ions during the reaction with *A. herba-alba* and *M. alba* extract was followed by UV–Vis spectroscopy and modification using spectrophotometry/96-well ELISA reader (ODs).

### Characterization of *A. herb-alba* and *M. alba* AuNPs using TEM

Antibacterial effect of biosynthetic AuNPs using well diffusion assay.

## Discussion

Antibiotics are widely used in animals at subtherapeutic doses to promote growth, and for the treatment of disease, resulting in the development of microbial resistance against these agents [31]. The current study determined the prevalence of virulence genes, namely, the beta-lactam resistance genes *bla_TEM_*, *bla_SHVm_*, *bla_OXA_*, and *bla_CTX−M_*, in *E. coli* and *Salmonella* spp. isolated from nasal and fecal swabs and milk samples of cattle, buffaloes, sheep, and goats with clinical signs including respiratory manifestations, pneumonia, diarrhea, and mastitis. It also evaluated the inhibitory effects of AuNPs synthesized using *A. herba-alba* and *M. alba* leaf extracts. Our results demonstrated that *E. coli* was present at a rate of 5.02% in nasal samples, 19.8% in fecal samples, and 37% in milk samples, as shown in Table-3. These results are in agreement with a previous study conducted by Amat [32]. *E. coli* is known to be a common opportunistic pathogen that causes several types of infection, causing diarrhea, mastitis, septicemia, and respiratory manifestations. In this work, the lowest positivity rates for *E. coli* were 3.8%, 5%, 5.2%, and 6.4% from nasal swabs of sheep, buffaloes, cattle, and goats, respectively (Table-3). This finding is in agreement with the previous studies [32,33] that found that *E. coli* was present at a low rate of 4.22% in healthy and diseased animals with respiratory signs. In addition, it was reported [34] that the rate of bacteria in the bovine nasopharyngeal tract was very low compared with the rates at other anatomical sites [35]. Another study explained the presence of virulence genes in extraintestinal *E. coli* [36]. The finding in the present work of the pathogenicity of *E. coli* isolated from nasal swabs of ruminants with respiratory signs may be associated with the inhalation of a number of bacteria from the environment [37]. The findings of the current study (Table-3) indicate that *E. coli* was detected at the highest rates of 25/105 (23.8%) and 11/52 (21.1%) from fecal swabs of goats and cattle with diarrhea, while *Salmonella* spp. were identified at the highest rate of 14/52 (26.9%) from fecal samples of cattle. These findings are in agreement with a previous study of Das *et al.*, [38] who found *E. coli* at a rate of 30% in cattle in Egypt but conflict with the results with Zeedan *et al*. [39]. Moreover, in another study, *E. coli* was identified in both diarrheic sheep and goats (34.7% and 30.7%), while *Salmonella* species were found at a rate of 3.6% in sheep and 2.6% in goats in Saudi Arabia [40]. Furthermore, in fecal samples collected from cattle in India, it was reported as 19% [41].

The observed variations in the prevalence of pathogenic bacteria in ruminants with clinical respiratory signs, diarrhea, and mastitis may be due to the studies focusing on different geographic areas, along with variations in animal management, in agreement with the previous studies [40,42]. The highest rate of positivity for *E. coli* in fecal samples and milk rather than in nasal swab samples (Table-3) may be due to the commonly found pathogenic *E. coli* in animal feces and shedding in manure or dung into the soil, leading to intense contamination with bacteria [43]. The highest rates of positivity of *E. coli* were 16/36 (44.4%) and 31/75 (41.3%) from milk samples of buffaloes and goats, respectively, as shown in Table-3, in agreement with many previous studies [44-46]. The inappropriate treatment of animal disease may thus cause the transmission of *E. coli* and *Salmonella* spp. The misuse of antibiotics in the veterinary field to prevent and control bacterial diseases in animals may have led to the development of resistance against these agents [47].

As shown in Table-4, *E. coli* and *Salmonella* spp. were 100% resistant to Am and Ax. In addition, both isolates were highly resistant to CX and Am/SAM, at rates of 86.4% and 81.8% for *Salmonella* spp. and 87.4% and 82.5% for *E. coli*. Meanwhile, both isolates were highly susceptible to IMP, cefotaxime, LEV, CIP, gentamicin, and amikacin, which are in agreement with the previous reports [38,48]. Isolated MDR *E. coli* with resistance to Am, Ax, Ax-clavulanic acid, and cefotaxime harbor the *bla*_TEM_, *bla_CTX_*, and *bla*_KPC_ resistance genes in Egypt. In addition, in a previous study [49], *Salmonella* spp. were isolated from livestock animals in South Africa with high rates of resistance to Am and Ax-clavulanate (64% and 63%, respectively). Am and Ax are intensively used for treating different animal infections in cattle, sheep, and goats [50].

**Table 4:** Antimicrobial susceptibility pattern of *E. coli* and *Salmonella* spp. isolated from clinical samples of different animals.

Antibiotic class	Antibiotic	*Salmonella* spp. (n=66)		*E. coli* (n=183)	
	
		*S*	*R*	*S*	*R*
Beta-lactams	Ax (25 μg)	0% (0)	100% (66)*	0% (0)	100% (183)A
	Am (10 μg)	0% (0)	100% (66*)	0% (0)	100% (183)B
	Fox (30 μg/disk)	13.6% (9)	86.4% (57)	12.56% (23)	87.4% (160)
	IMP (10 μg/disk)	87.9% (58)	12.2% (8)	93.5% (171)	6.5% (12)
	CTX (30 μg)	84.8% (56)	15.2% (10)	84% (154)	15.8% (29)
	SAM (20 μg)	18.2% (12)	81.8% (54)	17.48% (32)	82.5% (151)
Quinolones	LEV (5 μg)	90.9% (60)	9.1% (6)	96.1% (176)	3.8% (7)
	CIP (5 μg)	89.4% (59)	10.6% (7)	91.2% (167)	8.75% (16)
Aminoglycosides	CN (10 μg)	81.8% (54)	18.2 (12)	87.4% (160)	12.56% (23)
	AK (30 μg)	83.3% (55)	16.7% (11)	93,.4% (171)	6.55% (12)

Ax=Amoxicillin, Am=Ampicillin, Cx=Cefoxitin, Fox, IMP=Imipenem, CTX=Cefotaxime, AM/SAM: Ampicillin/sulbactam, LEV=Levofloxacin, CIP=Ciprofloxacin, CN=Gentamicin, AK=Amikacin, MET=Methicillin,

* *Salmonella* spp. isolates (n=66) (100% R)=(42 highly resistant isolates+24 isolates with low resistance) A: *E. coli* isolates (n=183) (100% R)=(113 highly resistant isolates+70 isolates with low resistance), *E. coli=Escherichia coli*

On farms, antibiotics tend to be administered to all animals during the treatment of different diseases, which promotes antibiotic resistance and has harmful effects on the intestinal microflora of healthy animals. Other antibiotics such as fluoroquinolones and beta-lactams are used as growth promoters on farms, leading to bacterial resistance in *Salmonella* spp. and *E. coli* [49-51]. Therefore, antibacterial agents should only be used in the veterinary and health sectors following accurate diagnoses, along with the routine application of AST [52]. Overall, in this study, resistance genes were found in *E. coli* at a rate of 61.2% and in *Salmonella* at 63.6%, as shown in Figures-1 and 2, which is in agreement with a previous study [53] demonstrating rates of resistant *E. coli* and *Salmonella* spp. of 60% and 50%, respectively, in the developing countries, they showed a resistance rate >50%. Among the resistance genes, the highest rate was found for *bla_TEM_* (21/81, 25.9%) in *E. coli* fecal swabs, while the lowest was for bla*_SHV_* (8/81, 9.8%) in *E. coli* nasal swabs, as shown in Figures-1 and 2. The high resistance may be due to the excessive use of antibiotics, in agreement with a previous study of Ogunrinu *et al*. [54]. The prevalence of *bla_CMY-2_* was 38.88% by Dong *et al*. [55]. The high prevalence of beta-lactam degrading genetic determinants may be due to the widespread use of extended beta-lactams such as Am and Ax for treating infections in cattle [48,55]. A possible explanation for the findings is that the feeding of animals on foods mixed with antibiotics as growth promoters has increased the rate of bacterial resistance [49]. *bla_CMY-2_* is involved in cell wall synthesis and membrane transport activity. The *bla_TEM-1_* and *bla_CMY-2_* resistance genes in bacteria cause the degradation of beta-lactams and extended beta-lactams, which could explain their association. *bla_TEM-1_* and *bla_CMY-2_* are located on transferable plasmids [57].

Antibiotic resistance is mediated by genetic elements through different mechanisms, leading to the escape of bacteria from the effects of antibiotics [58]. A previous study by Awosile [59] found that the beta-lactam (*bla_TEM-1_* and *bla_CMY_-*_2_) resistance genes encode enzymes that degrade beta-lactam antibiotics by hydrolyzing the beta-lactam ring. This is in agreement with our study that showed phenotypic resistance to beta-lactam antibiotics in *E. coli* and *Salmonella* spp. through the *bla_TEM_*, *bla_SHV_*, *bla_OXA_*, and bla*_CTX−M_* resistance genes.

The successful biosynthesis of *A. herba-alba* and *M. alba* AuNPs after adding a mixture of *A. herba-alba* and *M. alba* leaf aqueous extracts was indicated by a change in solution color from yellow to purple/dark red and dark brown, signifying AuNP formation (Figure-3). This was due to phytochemical substances in the aqueous extract, which are responsible for the creation of a coating on the AuNPs, as described previously by Abdel-Kareem and Zohri [60].

**Figure-4:**
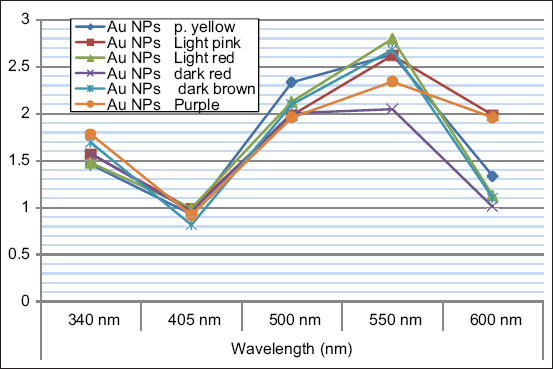
Ultraviolet spectra for *Artemisia herb-alba* and *Morus alba* AuNPs of different sizes and colors in colloidal solution. Strong resonance at 520-570 nm is clearly seen and arises due to the excitation of surface plasmon vibrations in the gold nanoparticles. The formation of gold nanoparticles (AuNPs) was confirmed using an ultraviolet-visible spectrophotometer at a wavelength of 520-570 nm based on surface plasmon resonance. The findings showed that the concentration and utilization of *A. herb-alba* and *M. alba* leaf extracts facilitated the production and strongly affected the optical properties of the AuNPs.

**Figure-5:**
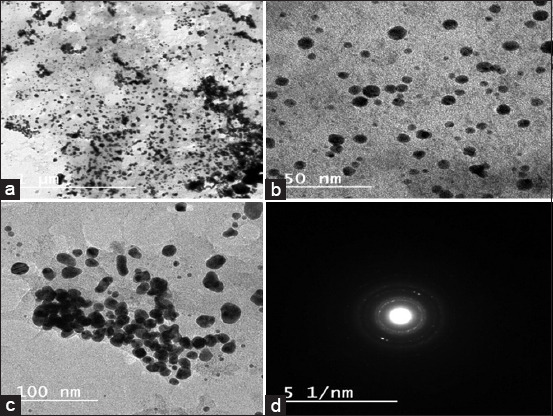
TEM micrographs of *Artemisia herba-alba* and *Morus alba* gold nanoparticles (AuNPs) of different sizes and shapes. A histogram of their distribution is shown in (a), with colloidal AuNPs being small and spherical, while aggregations have an average size of ~10. (b) The agglomeration of AuNPs was lower than the last one and the particle size was between 12 and 20 nm, showing almost spherical and separated particles. (c) Particle sizes were between 20 and 42 nm, with a rod-like appearance. The results suggest that the appearance of smaller particle size synthesized with large size nanoparticles is due to mixing aqueous extracts of *A. herb-alba* and *M. alba*.

**Figure-6:**
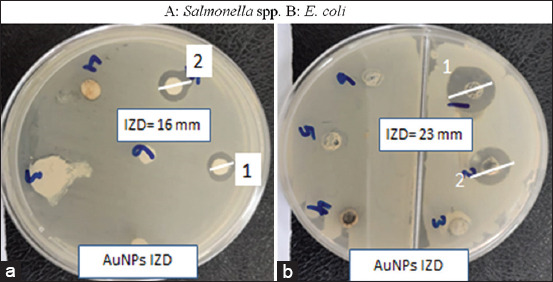
Evaluation of the inhibitory effect of *Artemisia herba-alba* and *Morus alba* gold nanoparticles (AuNPs) by well diffusion test. (a) *Salmonella* spp. and (b) *Escherichia coli* showing inhibition zone diameter of 16 and 23 mm, respectively.

**Figure-7:**
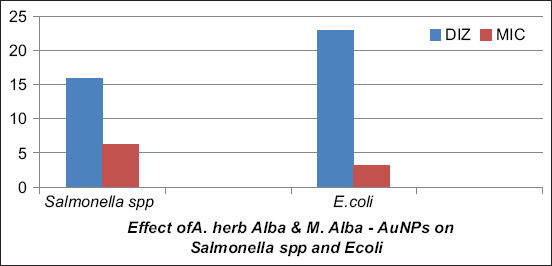
The inhibitory effect of biosynthetic *Artemisia herb-alba* and *Morus alba* gold nanoparticles (AuNPs), showing antibacterial activity against isolated bacteria. The inhibitory effects (IZD and MIC) of *A. herba-alba* and *M. alba* AuNPs on *Salmonella* spp. were 16 mm and 6.25 g/mL, and those on *Escherichia coli* were 23 mm and 3.125 g/mL, respectively. IZD: Inhibition zone diameter, MIC: Minimal inhibitory concentration.

The formation of AuNPs was confirmed by UV spectrophotometry at a wavelength of 520-570 nm based on surface plasmon resonance (SPR), as shown in Figure-4, and reported by Sundararajan and Kumari [61]. Synthesized AuNPs produced using *Pogostemon benghalensis* showed local SPR bands at 535-538 nm and 510-560 nm. In addition, the AuNPs were further characterized by TEM and micrography, demonstrating that they were small and spherical, with small and large aggregations and an average size of ~10-42 nm, as shown in Figure-5a-c. The spherical AuNPs can form at the beginning of the reaction, which then aggregates together due to the presence of a large amount of reducing agents in the biomass. This is in agreement with a previous study by Sundararajan and Kumari [60] in which different sizes of *Pityriasis alba* AuNPs were prepared and shown to have average sizes of 20.65 and 36.05 nm. This finding is also in agreement with the previous studies [61,62] that found that phytochemicals can effectively stabilize smaller AuNPs with an average size of 7 nm. As shown in Figure-5d, AuNPs were confirmed by selected area electron diffraction analysis. The micrograph showed spherical AuNPs in clear lattice fringes with bright circular rings corresponding to 5 to 1 nm planes. Different sets of spots could be identified from this diffraction pattern, as shown in Figure-5d. AuNPs similarly showed strong intensity at the 5-50 nm plane, with four concentric rings being observed in the AuNPs, indicating their crystalline nature. *A. herba-alba* and *M. alba* AuNPs had the maximum inhibitory effects on *E. coli* and *Salmonella* spp. with highest inhibition zone diameter and minimum inhibitory concentrations of 23 mm and 3.125 g/mL, respectively, for *E. coli*. This may have been due to the thin wall of the peptidoglycan layer in Gram-negative bacteria, which allow AuNPs to easily enter the cytoplasm, as shown in Figures-6 and 7. These results are in agreement with the previous studies [62,63] in which it was described that AuNPs have antibacterial activity. The antibacterial effects of *A. herba-alb*a and *M. alb*a AuNPs with different concentrations were higher than *A. herba-alb*a and *M. alb*a leaf extract against MDR *E. col*i and *Salmonell*a spp., as shown in Table-5. This is the first study on the biosynthesis of AuNPs using a mixture of *A. herba-alba* and *M. alba* leaf extracts, the effects of which may be due to their flavonoids, tannins, and polyphenolic compounds. This composition can explain their strong reducing activity to promote the formation of NPs. Finally, *A. herba-alba* and *M. alba* AuNPs have antibacterial effects against MDR *E. coli* and *Salmonella* spp. that are resistant to traditional antibiotics.

**Table 5:** Growth of bacteria in different concentrations of plant extracts and biosynthetic AuNPs

Name	10 μg/mL	20 μg/mL	40 μg/mL	60 μg/mL	80 μg/mL
*A. herb-alba* and *M. alba*	-	-	-	+	+
*A. herb-alba* and *M. alba* AuNPs	+	+	+	+	+
+ve control*	+	+	+	+	+
-ve control**	-	-	-	-	-

* Positive control (+ve)=Color change (red to pale yellow) indicating growth of *E. coli* and *Salmonella*,

** Negative control (−ve)=No change in color (red) indicating the absence of growth of *E. coli* and *Salmonella*. *E. coli=Escherichia coli, A. herba-alba=Artemisia herba-alba*, *M. alba=Morus alba,* AuNPs=Gold nanoparticles

## Conclusion

Bacterial resistance is one of the most important threats to animal health in the 21^st^ century, with bacterial cells developing resistance to one or more different antibiotics. Our study demonstrated the prevalence of MDR *E. coli* and *Salmonella* spp. isolated from different ruminants suffering from respiratory signs, diarrhea, and mastitis. The total rate of resistance to beta-lactam antibiotics in *E. coli* was 61.2%, while that in *Salmonella* was 63.6%. AuNPs were successfully biosynthesized using a mixture of aqueous leaf extracts of *A. herba-alba* and *M. alba*. They exhibited a particle size of 10-42 nm and exerted antibacterial effects on MDR *E. coli* and *Salmonella* spp. Alongside efforts to overcome the increasing rate of MDR bacteria in ruminants through several options, including improved animal hygiene and sanitation, as well as rapid and accurate diagnosis, NPs as alternative therapeutic agents are vital for effective treatment. Finally, NPs are the most promising strategy to overcome microbial drug resistance.

## Authors’ Contributions

AMA, AAA, ESI, and GSGZ: Designed the experiment and carried out the laboratory work. AAG: Contributed to the laboratory work and participated in drafting the manuscript. All authors read and approved the final manuscript.
